# Preparation and Performance Evaluation of CO_2_ Foam Gel Fracturing Fluid

**DOI:** 10.3390/gels10120804

**Published:** 2024-12-07

**Authors:** Yan Gao, Jiahui Yang, Zefeng Li, Zhenfeng Ma, Xinjie Xu, Ruiqiong Liu, Xin Li, Lixiao Zhang, Mingwei Zhao

**Affiliations:** 1 Changqing Downhole Technology Company, Chuanqing Drilling Engineering Company, China National Petroleum Corporation, Xi’an 710016, China; gaoyan029@cnpc.com.cn (Y.G.); cj_yangjiah@cnpc.com.cn (J.Y.); cj_lizef@cnpc.com.cn (Z.L.); 2 Key Laboratory of Unconventional Oil & Gas Development, China University of Petroleum (East China), Ministry of Education, Qingdao 266580, China; xuxinjie1028@163.com (X.X.); 19836391335@163.com (R.L.); lixindesuzi@163.com (X.L.); 18054340162@163.com (L.Z.)

**Keywords:** unconventional oil and gas resources, CO_2_ foam gel fracturing fluid, sand-carrying performance, high-temperature shear resistance

## Abstract

The utilization of CO_2_ foam gel fracturing fluid offers several significant advantages, including minimal reservoir damage, reduced water consumption during application, enhanced cleaning efficiency, and additional beneficial properties. However, several current CO_2_ foam gel fracturing fluid systems face challenges, such as complex preparation processes and insufficient viscosity, which limit their proppant transport capacity. To address these issues, this work develops a novel CO_2_ foam gel fracturing fluid system characterized by simple preparation and robust foam stability. This system was optimized by incorporating a thickening agent CZJ-1 in conjunction with a foaming agent YFP-1. The results of static sand-carrying experiments indicate that under varying temperatures and sand–fluid ratio conditions, the proppant settling velocity is significantly low. Furthermore, the static sand-carrying capacity of the CO_2_ foam gel fracturing fluid exceeds that of the base fluid. The stable and dense foam gel effectively encapsulates the proppant, thereby improving sand-carrying capacity. In high-temperature shear tests, conducted at a shear rate of 170 s^−1^ and a temperature of 110 °C for 90 min, the apparent viscosity of the CO_2_ foam gel fracturing fluid remained above 20 mPa·s after shear, demonstrating excellent high-temperature shear resistance. This work introduces a novel CO_2_ foam gel fracturing fluid system that is specifically tailored for low-permeability reservoir fracturing and extraction. The system shows significant promise for the efficient development of low-pressure, low-permeability, and water-sensitive reservoirs, as well as for the effective utilization and sequestration of CO_2_.

## 1. Introduction

Low-permeability oil and gas reservoirs account for a significant proportion of China’s total proven reserves, representing a critically important category of hydrocarbon resources for the oil and gas industry [1]. In recent years, these resources have attracted increasing attention from researchers and industry experts, underscoring their vital role in the exploration and development of oil and natural gas reserves [2,3]. However, the extraction of low-permeability oil and gas resources presents substantial challenges due to the inherent characteristics of these reservoirs, including low permeability, low porosity, poor reservoir quality, and significant heterogeneity [4].

Hydraulic fracturing is a critical industrial technique in oil and gas engineering, designed to enhance production and injection efficiencies. It has undergone extensive development and is widely applied across various types of oil and gas reservoirs, particularly those characterized by low permeability [5,6]. The process involves injecting working fluids into subterranean formations to initiate fractures, followed by the introduction of proppants to improve the inflow capacity of these fractures [7]. This enhances the permeability of reservoirs, leading to increased oil and gas production. Fracturing fluid, as a key component of this technology, serves as the operational medium during the fracturing process. The performance characteristics of the fracturing fluid are closely linked to its suitability for different geological formations [8,9]. Water-based fracturing fluids are the most commonly used due to their advantages, including high viscosity and rapid gel breakage. However, conventional water-based fracturing fluids are less effective in low-pressure, low-permeability, and water-sensitive reservoirs [10,11,12,13]. This limitation arises from the interaction between formation minerals and water, which can cause clay swelling and, consequently, a reduction in reservoir permeability [14,15].

Compared to traditional water-based fracturing fluids, foam gel fracturing fluids demonstrate superior applicability in low-pressure and low-permeability oil and gas reservoirs. These fluids offer numerous advantages, including minimal reservoir damage, enhanced oil recovery, rapid and complete gel breakage, low filtration loss, appropriate viscosity, reduced water consumption during operations, and a notable cleaning effect [16,17]. Foam gel fracturing fluids are generally classified into two types: CO_2_ foam gel fracturing fluids and N_2_ foam gel fracturing fluids. CO_2_ foam gel fracturing fluids use CO_2_ as the dispersed phase within the gaseous component, while the liquid phase is similar to traditional water-based fracturing fluids, consisting of water mixed with various additives. Compared to N_2_ foam gel fracturing fluids, CO_2_ foam gel fracturing fluids present several distinct advantages [18,19,20,21,22,23,24]: (1) CO_2_, as a high-density gas, provides exceptional foam stability, characterized by high density, robust stability, and low viscosity. This results in high hydrostatic pressure and low ground construction pressure, reducing pipeline friction loss. (2) CO_2_ foam gel fracturing fluids employ a range of mechanisms to enhance oil recovery, such as viscosity reduction, energy enhancement, anti-expansion, resistance reduction, and drainage assistance. These mechanisms mitigate the adverse effects on reservoir permeability, leading to superior oil recovery performance. (3) The lower pH of CO_2_ in foam gel fracturing fluids helps inhibit clay swelling, thereby reducing formation damage. (4) Carbon capture, utilization, and sequestration (CCUS) technology has emerged as a key strategy for ensuring national energy security and meeting CO_2_ emission reduction targets. The use of CO_2_ foam gel fracturing fluids in the petroleum industry not only contributes to these objectives but also provides valuable insights into CO_2_ sequestration and adsorption processes. However, many current CO_2_ foam gel fracturing fluids are hindered by challenges such as complex preparation processes, low system viscosity, and limited proppant-carrying capacity [25,26,27,28].

To address these limitations, this work introduces a novel CO_2_ foam gel fracturing fluid system which incorporates a domestically produced thickening agent CZJ-1, a foaming agent YFP-1, and CO_2_. This system demonstrates excellent foaming properties, foam stability, and robust sand-carrying capacity. Through optimization based on experimental conditions, the optimal dosages of the foaming agent and thickening agent were determined. Furthermore, the optimized formulation was evaluated for temperature resistance, shear resistance, and static sand-carrying performance, providing a solid foundation for the fracturing of low-permeability oil and gas reservoirs. The optimized fracturing fluid formulation not only enhances the temperature and shear resistance of the system but also exhibits impressive static sand-carrying performance. This work presents a simple, efficient, foam-stabilized, and sand-carrying system designed for the fracturing of low-permeability reservoirs, offering valuable insights and guidance for the fracturing of low-pressure, low-permeability reservoirs and the capture and storage of CO_2_.

## 2. Results and Discussion

### 2.1. Construction of CO_2_ Foam Gel Fracturing Fluid

#### 2.1.1. Determination of the Amount of Foaming Agent

At a temperature of 30 °C and under standard atmospheric pressure, varying mass fractions of YFP-1 were incorporated into a 1.5 wt% CZJ-1 solution to prepare base fluids with different foaming agent concentrations. The base fluid was then aerated and foamed using the Waring Blender method to produce CO_2_ foam gel fracturing fluid. The foaming volume and foam half-life were carefully monitored, and the foam composite index was calculated according to the method outlined in the literature [29]. The experimental results are presented in Table 1.

The experimental results indicate that the foaming volume, foam half-life, and foam composite index values initially increase and then decrease as the mass fraction of YFP-1 increases at 30 °C and atmospheric pressure. This behavior can be attributed to the fact that an increase in the mass fraction of the foaming agent promotes the formation of a stable liquid film [30]. However, as the foaming agent concentration reaches a certain threshold, its effect on the foaming characteristics diminishes, and the thickening agent begins to play a dominant role. This leads to an increase in the viscosity of the foam gel fracturing fluid and strengthens the network structure of the foam gel, making further foaming more difficult. Notably, the foam half-life and foam composite index reach their maximum values at a YFP-1 concentration of 0.7 wt%, with the 0.5 wt% concentration following closely behind.

**Figure 1:**
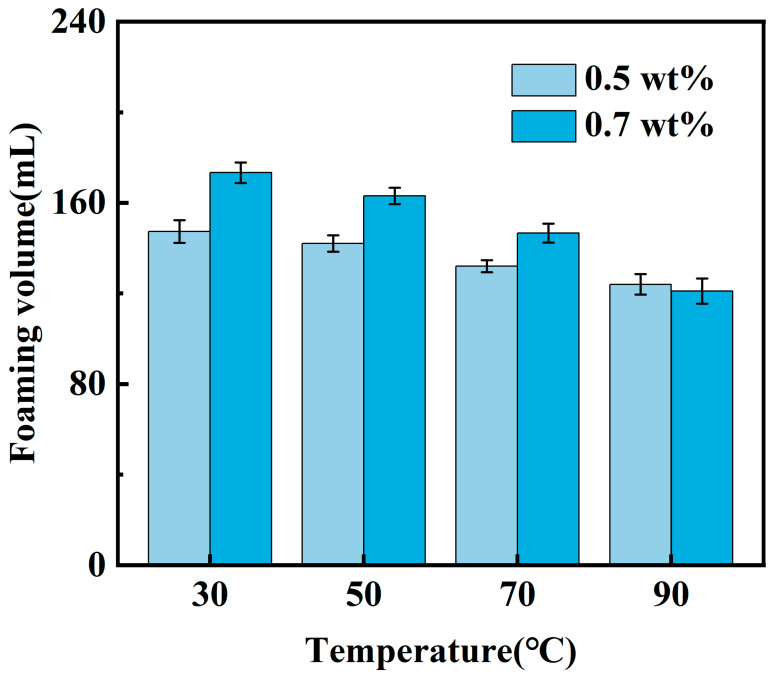
Foaming volume of foaming agent screening experiment.

**Figure 2:**
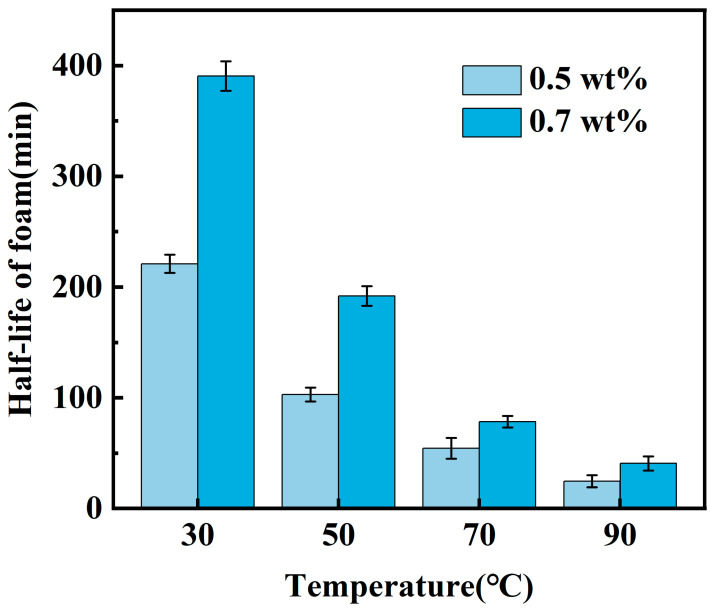
Half-life of foam in foaming agent screening experiment.

Although the fracturing fluid system exhibits a larger foam volume at a YFP-1 concentration of 1.5 wt% compared to concentrations of 0.5 wt% and 0.7 wt%, it suffers from a shorter foam half-life. This can be attributed to the excessively high concentration of the foaming agent, which reduces the influence of the thickening agent on the overall fracturing fluid system. As a result, the two components fail to synergize, making it difficult to form a stable network structure of the foam gel. Consequently, the two YFP-1 mass fraction concentrations (0.5 wt% and 0.7 wt%) that resulted in the highest overall foam performance were focused on for further investigation. The foam volume and foam half-life of the CO_2_ foam gel fracturing fluid were then evaluated under varying temperature conditions to assess its foaming performance and determine the optimal YFP-1 concentration. The experimental results are presented in Figure 1 and Figure 2.

The experimental results reveal that the foam half-life of the CO_2_ foam gel fracturing fluid system, formulated with two distinct mass fraction concentrations of YFP-1, decreases as the temperature increases. Notably, temperature exerts minimal influence on the foaming volume. This behavior can be attributed to the structure disruption of the thickening agent, which leads to a reduction in thickening viscosity at higher temperatures. When comparing the fracturing fluid systems with 0.5 wt% and 0.7 wt% YFP-1 concentrations, the system with 0.7 wt% YFP-1 exhibited greater stability, maintaining a foam half-life of 41 min at 90 °C. Based on the foaming performance, the optimal YFP-1 concentration was determined to be 0.7 wt%.

#### 2.1.2. Determination of the Amount of Thickening Agent

**Figure 3:**
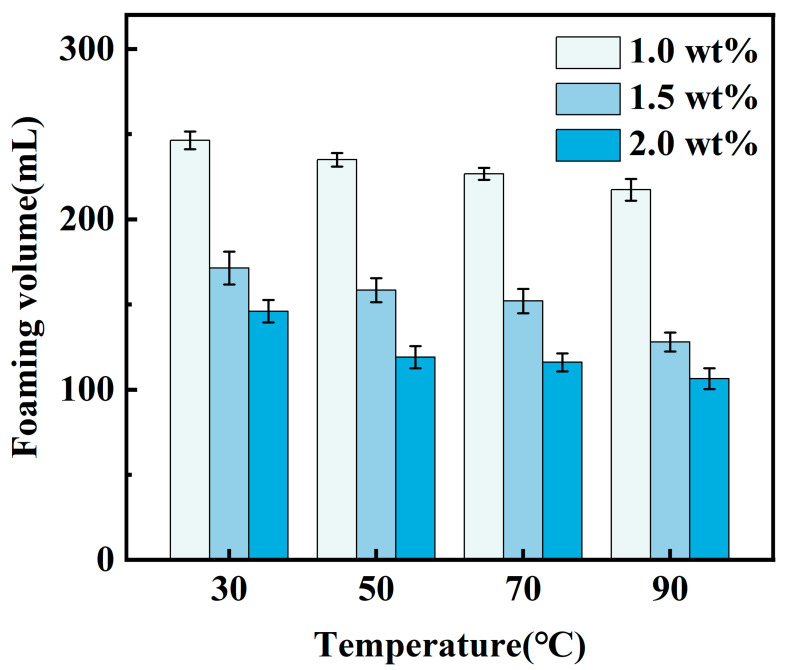
Foaming volume in thickening agent screening experiment.

**Figure 4:**
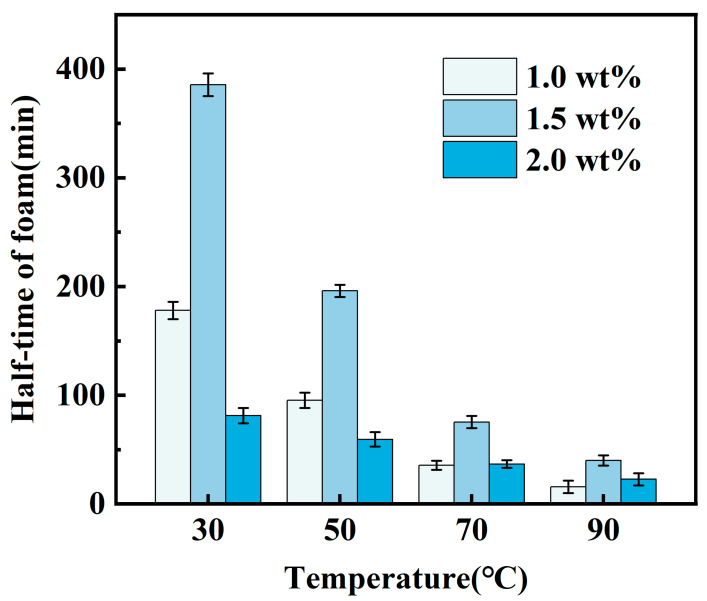
Half-life of foam in thickening agent screening experiment.

CO_2_ foam gel fracturing fluid systems were formulated under varying experimental temperature conditions using 1.0 wt%, 1.5 wt%, and 2.0 wt% CZJ-1 in combination with 0.7 wt% YFP-1. The foaming properties of these systems were then measured, and the results are presented in Figure 3 and Figure 4.

As the mass fraction of the thickening agent CZJ-1 increases, the foaming volume of the CO_2_ foam gel fracturing fluid system gradually decreases. Simultaneously, the foam half-life initially increases and then decreases, reaching its maximum at a concentration of 1.5 wt% CZJ-1. As the thickening agent concentration rises, the viscosity of the fracturing fluid system increases, which enhances foam stability. However, once the thickening agent concentration reaches its optimum level, the excessive viscosity hinders the effectiveness of the foaming agent YFP-1 as the stable network structure of the thickening agent solution impedes bubble formation.

To ensure that the CO_2_ foam gel fracturing fluid formulation meets the required specifications, comprehensive analyses were conducted on experimental data, including foaming volume, foam half-life, and the foam composite index for both the foaming agent and the thickening agent. Subsequently, considering both cost-effectiveness and performance, the optimal formulation for the CO_2_ foam gel fracturing fluid was established as follows: CO_2_ combined with 0.7 wt% YFP-1 and 1.5 wt% CZJ-1. All the subsequent experiments were carried out based on this optimized formulation.

### 2.2. Performance Evaluation of the CO_2_ Foam Gel Fracturing Fluid

#### 2.2.1. Static Sand-Carrying Performance

Sand-carrying performance, defined as the ability of foam to transport sand or rock fragments, is a critical performance metric in foam sand flushing and foam fracturing processes. This performance is typically assessed by the settling velocity of sand or rock particles within the foam, which serves as an indicator of the foam’s apparent viscosity and stability. For the CO_2_ foam gel fracturing fluid system, the goal is to maximize the sand-carrying capacity of both the base fluid and fracturing fluid to meet the requirements of various operational conditions. To evaluate this performance, the high-temperature visualized static sand-carrying sealed device and measuring cylinder are employed to test the sand-carrying capacity of the base fluid and CO_2_ foam gel fracturing fluid system under varying temperature and sand–fluid ratios.

**Figure 5:**
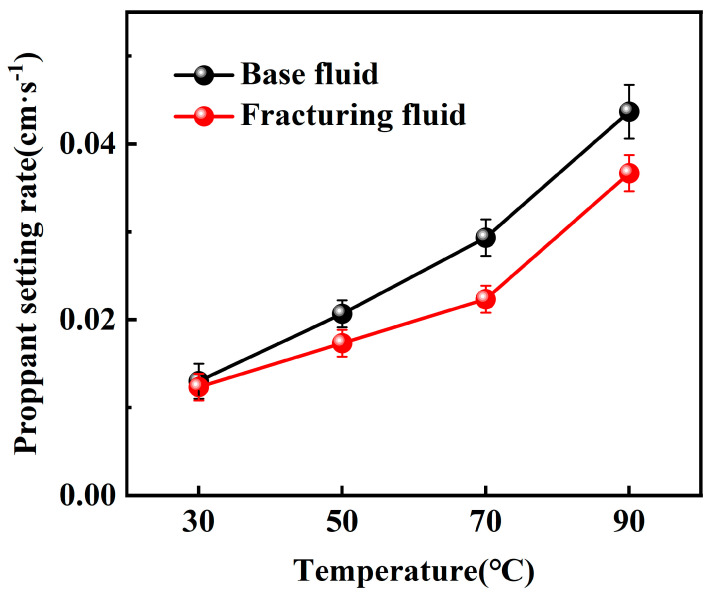
Variable temperature sand-carrying experiment with a high-temperature visualized static sand-carrying sealed device.

**Figure 6:**
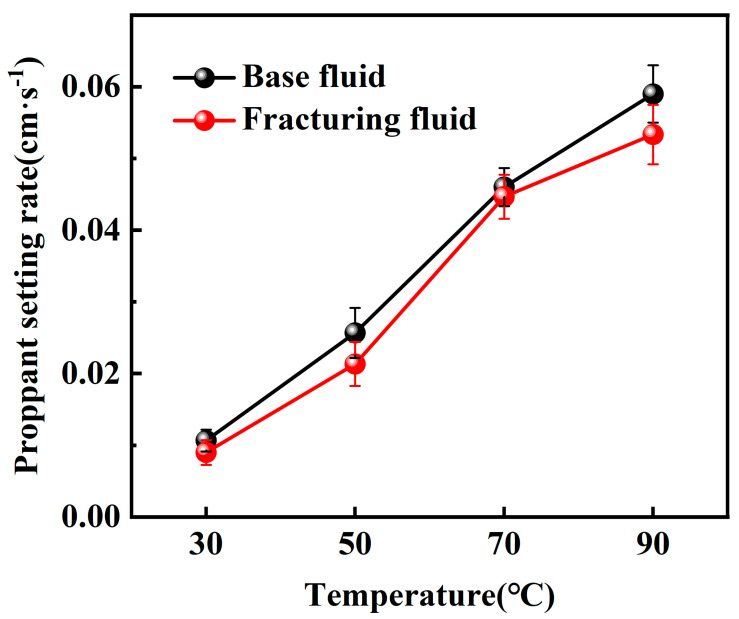
Variable temperature sand-carrying experiment with a measuring cylinder.

Static sand-carrying tests were first conducted with a constant sand–fluid ratio of 10%, while the temperature was varied across 30 °C, 50 °C, 70 °C, and 90 °C. The settling velocity of the proppant was then calculated, and the corresponding experimental results are presented in Figure 5 and Figure 6.

**Figure 7:**
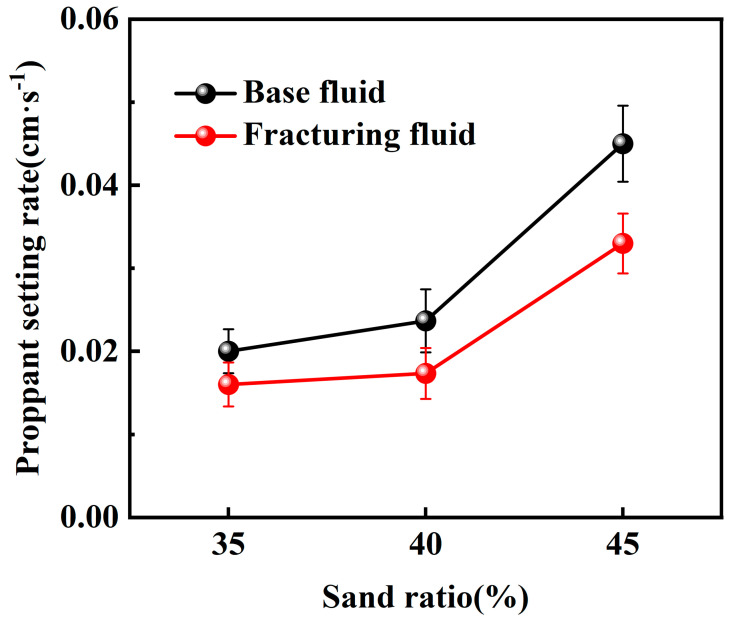
Sand-carrying experiment with a large sand ratio using a visualized static sand-carrying sealed device.

**Figure 8:**
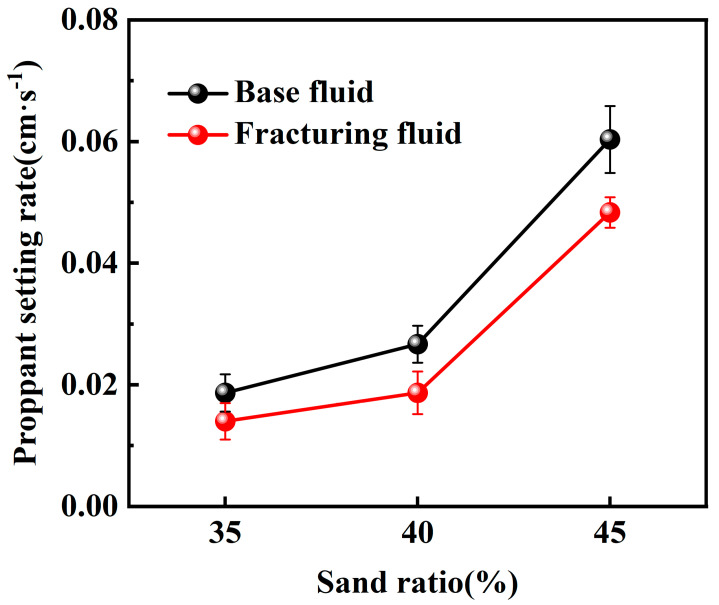
Sand-carrying experiment with a large sand ratio using a measuring cylinder.

According to the experimental results, when using the maximum particle diameter of commonly used 20–40 mesh proppant (0.9 cm), the proppant settling velocity in both the base fluid and the CO_2_ foam gel fracturing fluid remains below 0.06 cm·s^−1^, indicating a favorable range for allowable proppant settling. Furthermore, the settling velocity increases with the rise in temperature. Then, the static sand-carrying performance of the base fluid and the CO_2_ foam gel fracturing fluid was evaluated by varying the sand–fluid ratio at room temperature. The experimental results are presented in Figure 7 and Figure 8.

The experimental results indicate that the proppant settling velocity within the CO_2_ foam gel fracturing fluid meets the standard requirements, ranging from 0.008 to 0.8 cm·s^−1^, under room temperature conditions and with a high sand–fluid ratio. This finding suggests that the fracturing fluid system is capable of effectively carrying and transporting proppants at elevated sand ratios.

Under identical experimental conditions, the CO_2_ foam gel fracturing fluid system exhibits a distinct sand-carrying duration compared to the base fluid, with a notably slower settling velocity for the proppant. This is attributed to the differences in the sand-carrying mechanisms between the CO_2_ foam gel fracturing fluid and traditional water-based fracturing fluid, which relies solely on the viscosity of the liquid phase. In contrast, the CO_2_ foam gel fracturing fluid benefits from the coexistence of both gas and liquid phases. The liquid phase provides stable viscosity for carrying the proppant, while the gas bubbles encapsulate and support the proppant particles. The foam formed in the fracturing fluid has a stronger interface effect than that in water-based fracturing fluids [31]. The greater the interfacial film strength between the foam bubbles, the more stable, dense, and resilient the foam becomes, leading to an enhanced proppant-carrying capacity. As a result, this improves the uniform dispersion of proppant particles within the foam.

#### 2.2.2. Steady Shear Performance

**Figure 9:**
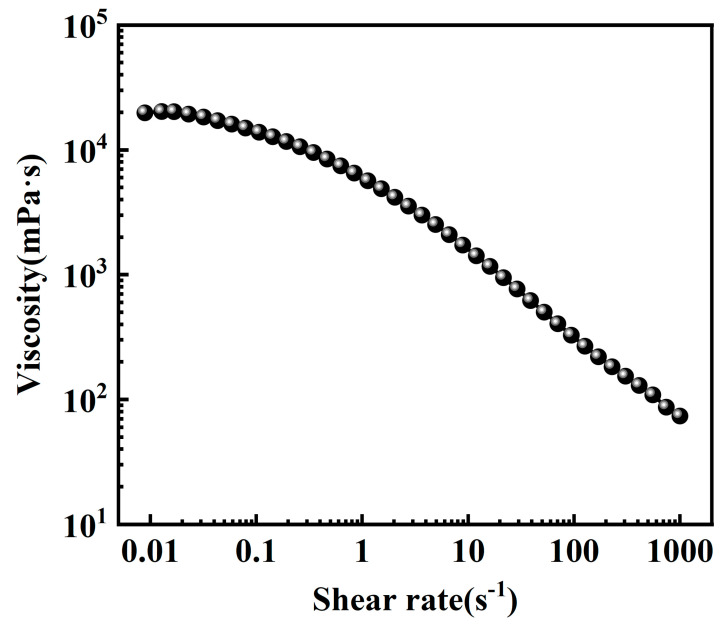
Steady shear curve of the CO_2_ foam gel fracturing fluid.

Figure 9 shows the variation in the viscosity of the CO_2_ foam gel fracturing fluid at different shear rates, demonstrating the characteristics of a non-Newtonian fluid. At low shear rates, the viscosity of the CO_2_ foam gel fracturing fluid remains relatively stable with minimal fluctuation. As the shear rate increases, the CO_2_ foam gel fracturing fluid exhibits typical non-Newtonian behavior, namely shear thinning. Nevertheless, even at a shear rate of 1000 s^−1^, the viscosity of the CO_2_ foam gel fracturing fluid remains at 74 mPa·s, indicating good shear resistance.

#### 2.2.3. Temperature and Shear Resistant Performance

**Figure 10:**
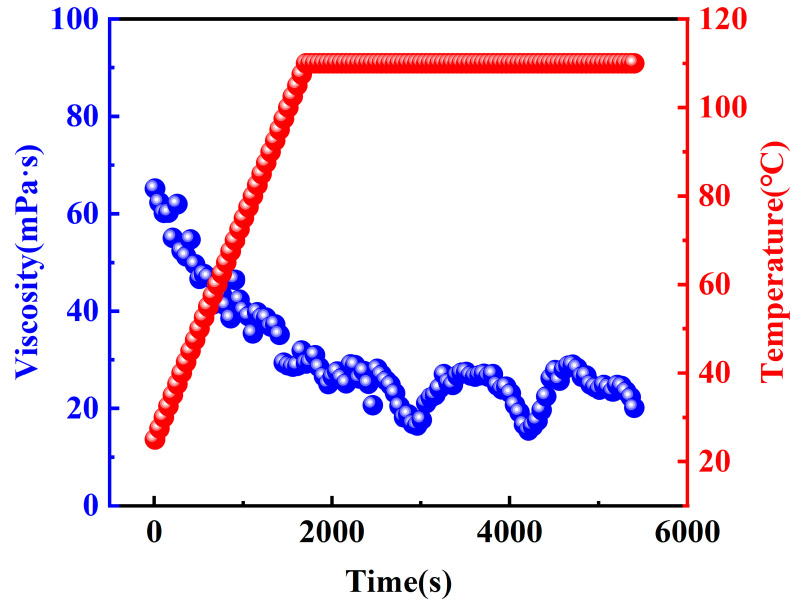
Test on temperature and shear resistance of base fluid.

**Figure 11:**
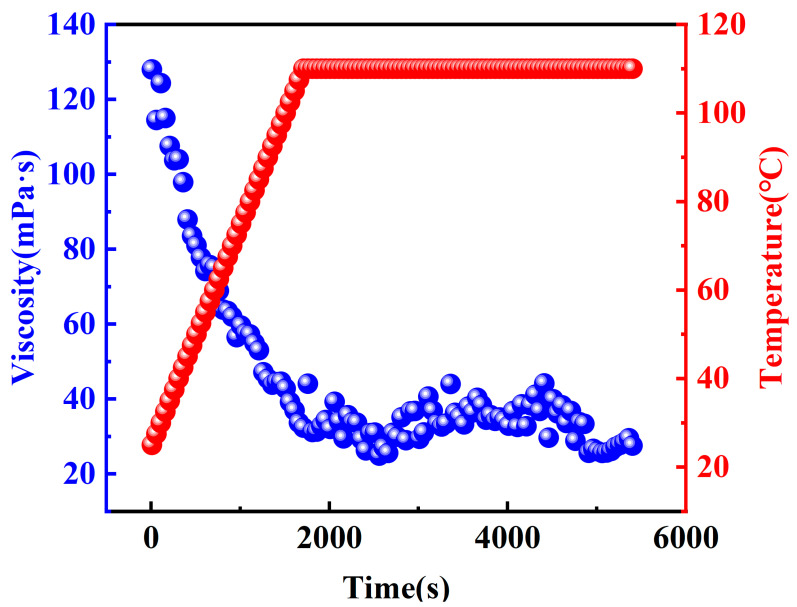
Test on temperature and shear resistance of the CO_2_ foam gel fracturing fluid.

To ensure that the fracturing fluid meets performance criteria, including foam stability, sand-carrying capacity, and seam-forming ability, it is essential for the CO_2_ foam gel fracturing fluid to maintain consistent viscosity under formation temperature conditions [32]. Additionally, the impact of foam on the temperature and shear stability of the fracturing fluid, both before and after foaming with CO_2_ gas, was further investigated. Consequently, the temperature and shear resistance performance of the optimized base fluid and fracturing fluid system were tested at an experimental temperature of 110 °C. The results of these tests are presented in Figure 10 and Figure 11.

The experimental results indicate that the apparent viscosities of both the base fluid and the CO_2_ foam gel fracturing fluid system gradually decrease during the heating and shearing process. Notably, during the subsequent constant-temperature shearing phase, which follows the heating process, the apparent viscosities of both systems tend to stabilize. Under identical experimental conditions, the viscosities of the CO_2_ foam gel fracturing fluid consistently exceed those of the base fluid. This difference is closely related to the incorporation of CO_2_ gas, as well as the stability and densification of the resulting foam gel structure.

The apparent viscosity of the base fluid decreases due to the differential disruption of the network structure of the thickening agent caused by heating and shearing. In the case of CO_2_ foam gel fracturing fluid, in addition to this mechanism, the expansion of CO_2_ gas further affects the foam system, leading to fluctuations in its apparent viscosity. These fluctuations result from the degradation of the foam film structure and the accelerated rates of foam film rupture and foam aggregation induced by heating and shearing [33]. These factors play a crucial role in the observed viscosity reduction. Under formation temperature conditions, the apparent viscosity of the fracturing fluid remained above 20 mPa·s at the end of the test, demonstrating that the fracturing fluid system maintained temperature resistance up to 110 °C. These results indicate strong temperature and shear resistance, meeting industry standards for fracturing fluids.

## 3. Conclusions

A CO_2_ foam gel fracturing fluid system has been developed with simple preparation conditions, making it well suited for application in low-pressure, low-permeability, and water-sensitive formations. The formulation of this system was optimized through a comprehensive evaluation of both the foaming volume and the foam half-life. The optimized formulation consists of 0.7 wt% foaming agent YFP-1, 1.5 wt% thickening agent CZJ-1, and CO_2_ gas.

The static sand-carrying performance of the optimized base fluid and CO_2_ foam gel fracturing fluid system was evaluated using a high-temperature visualized static sand-carrying sealed device and measuring cylinder under varying sand ratios and temperatures. Both the base fluid and the CO_2_ foam gel fracturing fluid system demonstrated a relatively slow proppant settling velocity and good sand-carrying performance across different experimental conditions, including variations in temperature and proppant dosage. Under identical experimental conditions, the proppant settling velocity of the CO_2_ foam gel fracturing fluid system was lower than that of the base fluid, indicating that the incorporation of CO_2_ and the formation of foam gel enhanced the interface effects in the fracturing fluid system. The dense and stable foam gel structure was able to encapsulate and support the proppant more effectively than the base fluid alone.

Under the experimental conditions of a shear rate of 170 s^−1^ and a temperature of 110 °C, the apparent viscosity of the CO_2_ foam gel fracturing fluid system remained above 20 mPa·s even after 90 min of shearing, indicating excellent temperature and shear resistance. Moreover, under the same experimental conditions, the apparent viscosity of the CO_2_ foam gel fracturing fluid system was found to be higher than that of the base fluid. This suggests that the foam generated within the fracturing fluid system has a stabilizing effect on the base fluid, thereby enhancing its viscosity.

## 4. Materials and Methods

### 4.1. Materials

In this work, sodium dodecyl benzene sulfonate (YFP-1, 95.0 wt%) was purchased from Shanghai Aladdin Reagent Co., Ltd., Shanghai, China. The thickening agent is a CO_2_-responsive surfactant (CZJ-1) supplied by China National Petroleum Corporation Chuanqing Drilling and Exploration Engineering Company, Xi’an, China. The CO_2_ gas, with a purity greater than 99 wt%, was sourced from Qingdao Deyi Gas Company, Qingdao, China. The proppant was ceramic granules, with a size of 20–40 mesh. Deionized water, prepared in the laboratory, was employed in the formulation.

### 4.2. Preparation of the CO_2_ Foam Gel Fracturing Fluid

**Figure 12:**
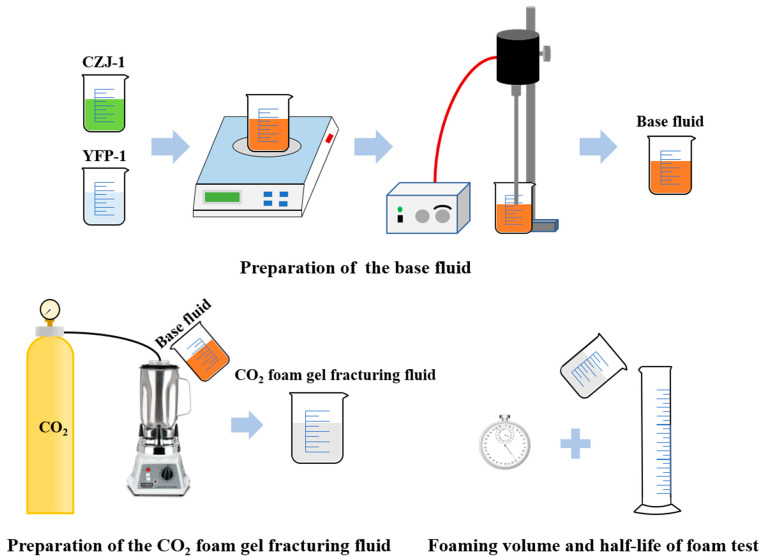
Schematic diagram of the CO_2_ foam gel fracturing fluid preparation.

The CO_2_ foam gel fracturing fluid was prepared using the Waring Blender method [34], as shown in Figure 12. First, 100 mL of the prepared base fluid was poured into the measuring cup of the Waring Blender. The CO_2_ gas source was then connected, and CO_2_ was injected at a controlled flow rate for 3 min to ensure that the base fluid remained in a CO_2_ environment throughout the process. The rotational speed of the Waring Blender was gradually adjusted until the rotating blades were just visible. The gas–liquid mixture in the measuring cup was stirred for 3 min. Afterward, the resulting CO_2_ foam gel fracturing fluid was poured into a 500 mL measuring cylinder. The volume of the foam in the cylinder was recorded as the foaming volume of the foam gel fracturing fluid. The time required for the foam to decrease to half of the initial base fluid volume was measured with a stopwatch and recorded as the foam’s half-life.

Based on previous experimental experience, the initial mass fraction of the thickening agent was set at 1.5 wt%. The foaming agent concentration was then optimized based on the foaming volume and foam half-life of the fracturing fluids with varying concentrations of the foaming agent. Subsequently, at the optimal foaming agent concentration, the thickening agent concentration was optimized by evaluating the foaming volume and foam half-life of the fracturing fluids with different thickening agent concentrations, thereby determining the final formulation of the CO_2_ foam gel fracturing fluid.

### 4.3. Sand-Carrying Performance Test Experiment

#### 4.3.1. High-Temperature Visualization Static Sand-Carrying Experiment

**Figure 13:**
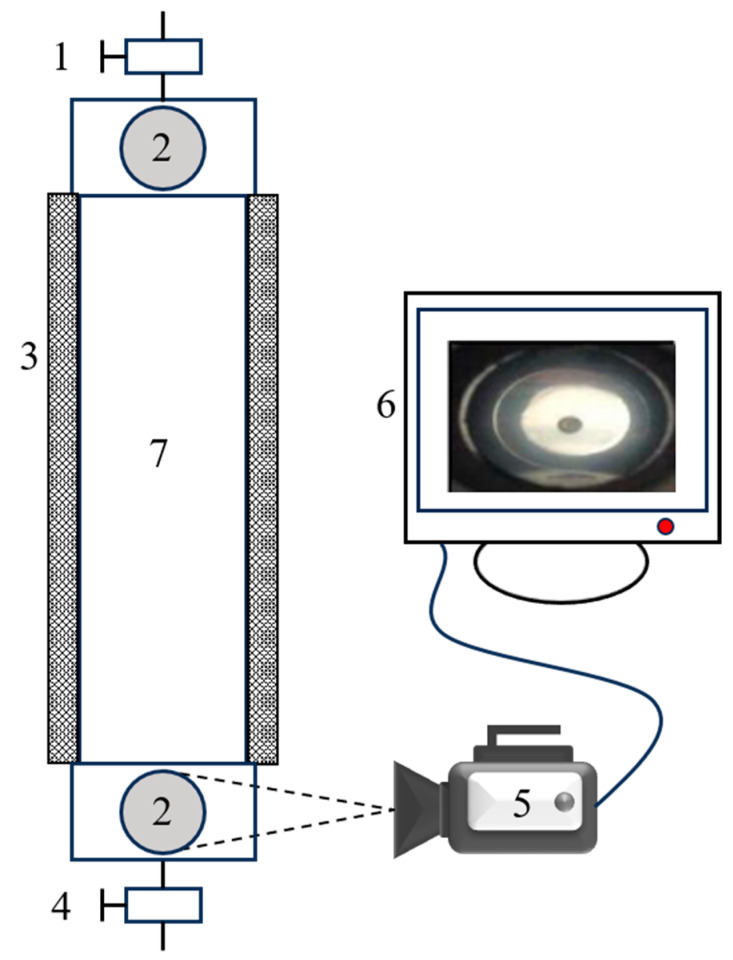
High-temperature visualized static sand-carrying sealed device: 1—fracturing fluid inlet valve; 2—transparent observation window; 3—temperature controlled cylinder; 4—pressure relief valve; 5—high-speed video camera; 6—data acquisition system; 7—inner cylinder.

A high-temperature visualized static sand-carrying sealed device was used to conduct static sand-carrying experiments on both the base fluid and the CO_2_ foam gel fracturing fluid system. The device includes a fracturing fluid inlet valve, a transparent observation window, a temperature-controlled cylinder, a pressure relief valve, a high-speed video camera, a data acquisition system, an inner cylinder, and other essential components, as illustrated in Figure 13. The experimental procedure involved preheating the inner cylinder of the device to the desired experimental temperature using the temperature control cylinder. A proppant with a specified sand–fluid ratio was then added to the inner cylinder, followed by the introduction of either the aged base fluid or the CO_2_ foam gel fracturing fluid system. After sealing both the inlet and outlet valves, the entire sand-carrying device was repeatedly inverted to ensure the thorough mixing of the proppant with the fracturing fluid. Following a period of equilibration, the device was inverted 180°, and a high-speed camera was used to record the time interval from the initial fall of the first ceramic proppant to the final fall of the last proppant. These data were subsequently used to calculate the average settling velocity of the proppants under various experimental temperatures and proppant dosages within the sand-carrying device.

#### 4.3.2. Static Sand-Carrying Measuring Cylinder Test

The base fluid or CO_2_ foam gel fracturing fluid, prepared under specified temperature conditions, was transferred into a beaker. The proppant, with a defined sand–fluid ratio, was then gradually and uniformly incorporated into the fluid while stirring continuously. Once the proppant was thoroughly mixed with the base fluid or CO_2_ foam gel fracturing fluid, the entire mixture was promptly transferred into a 500 mL measuring cylinder. This cylinder was then placed in a preheated thermostatic water bath set to the temperatures corresponding to the different experimental conditions. Using a stopwatch, the time required for all the proppant particles to settle to the bottom of the measuring cylinder was observed and recorded. Based on the recorded settlement times, the average settling velocity of the proppant was calculated for various experimental temperatures and different proppant dosages.

### 4.4. Temperature and Shear Resistance Test Experiments

A total of 50 mL of the prepared base fluid or CO_2_ foam gel fracturing fluid was placed into the rotating cylinder of the Haake Mars 60 rheometer (Karlsruhe, Germany). The temperature and shear resistance of both the base fluid and the CO_2_ foam gel fracturing fluid system were then evaluated using a PZ38 HA rotor. This evaluation was conducted at a constant shear rate of 170 s^−1^ and a temperature of 110 °C over a total duration of 90 min. This work focused on the effect of temperature variation on the apparent viscosity at the fixed shear rate, specifically monitoring the changes in apparent viscosity as a function of experimental temperature.

## Figures and Tables

**Table 1:** Foaming volume and half-life of foam with different amounts of YFP-1 at 30 °C.

YFP-1 (wt%)	Foaming Volume (mL)	Half-Life of Foam (min)	Foam Composite Index (mL·min)
0.3	120	196	23,520
0.5	148	230	34,040
0.7	173	394	68,162
0.9	140	197	27,580
1.5	195	119	23,205

## Data Availability

The original contributions presented in this study are included in the article. Further inquiries can be directed to the corresponding authors.
